# Maintenance of epigenetic landscape requires CIZ1 and is corrupted in differentiated fibroblasts in long-term culture

**DOI:** 10.1038/s41467-018-08072-2

**Published:** 2019-01-28

**Authors:** Emma R. Stewart, Robert M. L. Turner, Katherine Newling, Rebeca Ridings-Figueroa, Victoria Scott, Peter D. Ashton, Justin F. X. Ainscough, Dawn Coverley

**Affiliations:** 1 Department of Biology, University of York, York, YO10 5DD UK; 2 York Bioscience Technology Facility, University of York, York, YO10 5DD UK; 3 Present Address: Department of Genetics, University of Cambridge, Cambridge, CB2 3EH UK

**Keywords:** Epigenetics analysis, Epigenetics, Chromatin, DNA replication

## Abstract

The inactive X chromosome (Xi) serves as a model for establishment and maintenance of repressed chromatin and the function of polycomb repressive complexes (PRC1/2). Here we show that Xi transiently relocates from the nuclear periphery towards the interior during its replication, in a process dependent on CIZ1. Compromised relocation of Xi in CIZ1-null primary mouse embryonic fibroblasts is accompanied by loss of PRC-mediated H2AK119Ub1 and H3K27me3, increased solubility of PRC2 catalytic subunit EZH2, and genome-wide deregulation of polycomb-regulated genes. Xi position in S phase is also corrupted in cells adapted to long-term culture (WT or CIZ1-null), and also accompanied by specific changes in EZH2 and its targets. The data are consistent with the idea that chromatin relocation during S phase contributes to maintenance of epigenetic landscape in primary cells, and that elevated soluble EZH2 is part of an error-prone mechanism by which modifying enzyme meets template when chromatin relocation is compromised.

## Abstract (web summary)

The inactive X chromosome (Xi) is a model for establishment and maintenance of repressed chromatin and the function of polycomb repressive complexes. Here the authors show that Xi transiently relocates from the nuclear periphery during replication in a CIZ1-dependent manner, which plays a role in maintaining PRC-mediated repressed chromatin.

## Introduction

The inactive X chromosome (Xi) is a discrete unit of facultative heterochromatin that is selected for repression early in the development of female mammals as a means of equalizing X-linked gene dosage between the sexes^1^. *Xist* long noncoding RNA (LNCRNA) plays an essential role in the recruitment of chromatin modifying enzymes to Xi, and the progressive formation of a stable, heritable repressed state^2^. Detailed analysis shows that *Xist*-dependent polycomb recruitment is initiated by PRC1, and is dependent on the RNA-binding protein hnRNPK through its interaction with sequences encoded by *Xist* repeat B^3^. Later steps in the polycomb cascade result in the accumulation of PRC1-mediated H2AK119ub1 and PRC2-mediated H3K27me3 on Xi chromatin, which is then maintained through subsequent rounds of cell division^4^. CIP1/CDKN1A-interacting zinc finger protein 1 (CIZ1) is recruited to Xi by *Xist* during the earliest stages of X-inactivation dependent on sequences encoded by *Xist* repeat E^5,6^, though lack of overt embryonic phenotype in CIZ1 null mice suggest that there is no requirement for CIZ1 during these early stages of X-inactivation^5^. However, CIZ1 is required for retention of *Xist* at Xi in differentiated fibroblasts, and essential for its recruitment during lymphocyte activation in response to antigen stimulation in adult mice^5^, suggesting that it has a post-developmental function at Xi.

CIZ1 has been linked with the neurological disorders cervical dystonia^7^ and Alzheimer’s disease^8^, and with both paediatric^9^, and adult common solid tumours including lung, colon, liver and breast^10–13^, though no known underpinning molecular function convincingly links its role in these diverse human pathologies. Similarly, while a link with lymphocyte activation is established, the molecular mechanism that underpins its ability to guard against leukemias and lymphomas in mice is not understood^5,11,14^ Moreover, while enrichment at Xi in female cells is striking (Xi-CIZ1), CIZ1 protein also occupies nucleus-wide foci in male and female somatic cells (focal-CIZ1)^5^, and is elevated in post-replicative male germ cells^15^ suggesting that it has additional functions unrelated to the inactive X-chromosome.

In the present study, Xi serves as a well-defined model to probe the mechanism of action of CIZ1, and shows that CIZ1 is required to support a change in the preferred location of Xi, between the nuclear periphery and the nuclear interior, during a brief window coincident with Xi replication. In CIZ1 null fibroblasts, failure to internalize is accompanied by the loss of PRC1/2-mediated modification of Xi chromatin, and relaxation of control over PRC1/2 target genes across the genome. Crucially, S-phase internalization of Xi is not observed in fibroblasts in long-term culture, even if CIZ1 is present, suggesting that the process in which CIZ1 normally functions is fragile, and corrupted at some level in cell lines. Moreover, the loss of function in cell lines is accompanied by up-regulation and increased solubility of PRC2 catalytic subunit EZH2, and in CIZ1 null cells, partial reinstatement of chromatin modification at Xi. This raises the possibility that the mechanism by which modifying enzyme and target chromatin meet is not the same in primary cells and derived cell lines. The data support the idea that chromatin relocation during S phase plays a role in the maintenance of epigenetic state in primary differentiated cells.

## Results

### Interaction between CIZ1 and nuclear matrix at Xi in S phase

**Fig. 1:**
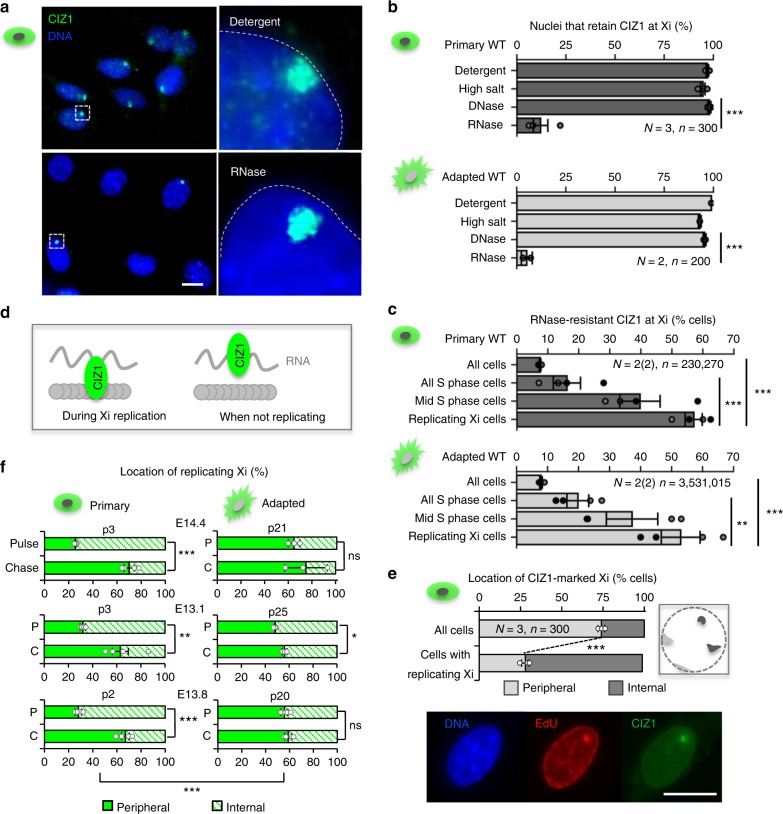
Xi location during S phase. **a** CIZ1 at Xi in cycling WT primary embryonic fibroblasts (PEFs), with and without extraction of RNA and associated proteins, detected with anti-CIZ1 antibody 1794 (green). DNA is blue, scale bar 10 microns. **b** Proportion of cells that retain CIZ1 at Xi (Xi-CIZ1) at the indicated steps after extraction, for three independent populations of WT PEFs (means ± SEM for 13.1, 13.8, 14.4 at passage 3–4, *n* > 100 for each step and each line. Significant differences are indicated (*t*-test). Similar results for culture-adapted derivative line 13.1 (passage > 20), and 3T3 cells is shown below, ±SDEV. **c** Proportion of cells with RNase-resistant Xi-CIZ1 after pulse-labelling with EdU to classify cells by S-phase stage (Supplementary Fig. 1). Upper graph shows mean data for WT primary cells 13.1 and 13.8 (passage 3–4), each in duplicate, ±SEM (*t*-test). Lower graph shows the same analysis for WT MEFs (13.1, passage > 20), and 3T3 cells, each in duplicate. **d** Illustration of transient attachment of Xi-CIZ1 (green) to non-chromatin, non-RNA assemblies (grey circles) during Xi replication. **e** Location of Xi-CIZ1 at the time of Xi replication (detected by 30 min pulse label with EdU) compared to the rest of the cell cycle. Location is classified as peripheral or internal (illustrated right). Means ± SEM for three WT PEFs (13.1, 13.8, 14.4 p3–4) are shown (*n* = 100 for each), with significant changes indicated (*t*-test). Images show a cell engaged in replication of Xi (EdU, red), co-stained with anti-CIZ1 1794 (green), DNA is blue, scale bar 10 microns. **f** Xi location (peripheral or internal) at the time of incorporation of EdU (pulse), or 30 min later (chase), analysed by *t*-test. Graphs show data ± SEM for three WT primary populations (13.1, 13.8, 14.4 p2–3), and three derived MEF lines (*p* > 20). EdU-Xi in primary WT cells shifts preferred location during the chase period, but remains unchanged in most adapted lines, compared by ANOVA. For all analyses *P ≤ 0.05, **P ≤ 0.01, ***P ≤ 0.001. *N* = independent cell lines, *n* = total nuclei scored. Individual replicates contributing to each mean are overlaid on bar charts, shaded according to cell line where necessary

Enzymatic removal of chromatin (DNase1) or exposure to elevated non-physiological salt concentrations (500 mM NaCl) have little effect on either Xi-CIZ1 or focal-CIZ1^5,16^, indicating that their location in the nucleus is not specified by association with chromatin. However, Xi-CIZ1 is sensitive to digestion with RNase in the majority of cells in a cycling population, indicating that attachment at Xi is by association with RNA^5^, most likely *Xist*^6,17^. We focus here on the small fraction of cells (3–7% depending on cell line and rate of cycling) that resist extraction with RNase so that CIZ1 remains anchored at Xi (Fig. 1a, b). This indicates additional or alternative interaction between CIZ1 and non-RNA, non-chromatin higher-order assemblies, consistent with a biochemically defined nuclear matrix (NM).

The low frequency of retention implies temporal control, which was supported by analysis in S phase cells. Incorporation of the nucleotide analogue 5-ethynyl-2ʹ-deoxyuridine (EdU) identified Xis engaged in DNA replication and showed that Xi replication occurs in mid S phase in most cells in the fibroblast populations studied here, though the timing of Xi replication appears to vary with cell type^18,19^ (Supplementary Fig. 1). When combined with immuno-staining for CIZ1, it also showed that CIZ1 is significantly more likely to be resistant to extraction at the time of Xi replication (Fig. 1c, Supplementary Fig. 1). In fact, over half of Xi-replicating cells retain CIZ1 at Xi after RNase digestion (Fig. 1d). This was evident in triplicate independent populations of primary embryonic fibroblasts (PEFs, defined here as passage 5 or earlier), and remained unchanged in all derived populations adapted to long-term growth in culture (referred to as mouse embryonic fibroblasts, MEFs). Thus, during Xi replication, Xi-CIZ1 undergoes transient interaction with a structure that is independent of chromatin or RNA.

### A shift in Xi location in primary cells but not cell lines

The location of replicating CIZ1-marked Xis was not the same in PEFs and MEFs. In primary cells, it was significantly more likely to be seen at locations that did not contact the nuclear periphery (Fig. 1e). To confirm the preferred position independently of CIZ1, we monitored the location of EdU-labelled Xis at the time of Xi replication/EdU incorporation (pulse) compared to its location 30 min later (chase), and relative to the nucleolar marker fibrillarin (Supplementary Fig. 1). This showed a greater likelihood of overlap with fibrillarin-enriched regions at the time of the pulse, compared to after a chase period, indicating a change in preferred location during S phase. Although Xi is located at the nuclear periphery most of the time, anchored via lamin B receptor^20^, these results are consistent with the very earliest descriptions of the Xi as occupying two distinct locations; the nuclear periphery or adjacent to nucleoli^21^.

Importantly, in three populations of primary cells isolated from independent WT embryos, this change in preferred location during S phase was readily monitored using a binary classification of peripheral or internal (P or I, Fig. 1f), but in culture-adapted lines derived from all three parent populations the shift in location was not evident (Fig. 1f), though replication of Xi took place in a similarly co-ordinated manner as a condensed EdU-labelled, CIZ1-marked entity. These data show that Xi replication is normally coincident with departure from the nuclear periphery, and suggest that the underlying mechanism for the change in preferred location is fragile and prone to degradation in culture-adapted cell lines. Moreover, it suggests that most cell lines likely do not represent the state of cells in the body with respect to chromatin dynamics, leading to questions about the functional relevance of the S-phase location change.

### CIZ1 is involved in PRC1/2 target gene regulation

**Fig. 2:**
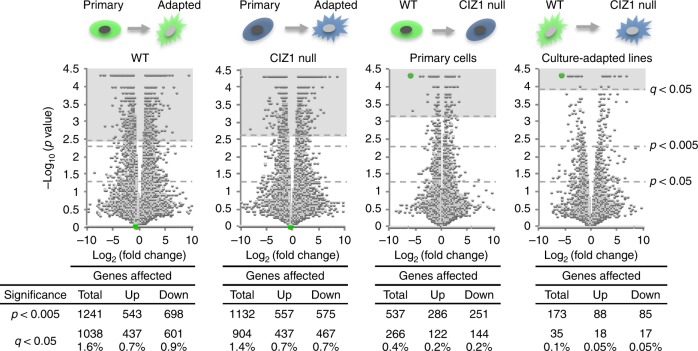
Effect of loss of CIZ1 and culture adaption on gene expression. Volcano plots showing mean fold change in transcript level (FPKM) against significance (*P*-value, calculated by cuffdiff) for all 65530 annotated transcription units assigned to mouse genome assembly GRCm38, derived from whole transcriptome RNA-seq of three primary WT (13.1 p4, 13.8 p4, 14.4 p4) embryonic fibroblast populations and culture-adapted cell lines derived from the same primary cells, and three primary CIZ1 null (13.15 p3, 13.17 p3, 14.2 p4) embryonic fibroblast populations and culture-adapted cell lines derived from the same primary cells. CIZ1 is indicated in green. The populations of cells being compared are indicated above each plot. Below, the number of transcription units affected at the indicated significance thresholds. Significantly affected gene lists (*q* < 0.05, calculated by cuffdiff) are in Supplementary data 1 (primary to culture-adapted comparisons for both genotypes), Supplementary data 4 (CIZ1-dependent genes in primary cells), Supplementary data 5 (CIZ1-dependent genes in culture-adapted cells)

**Fig. 3:**
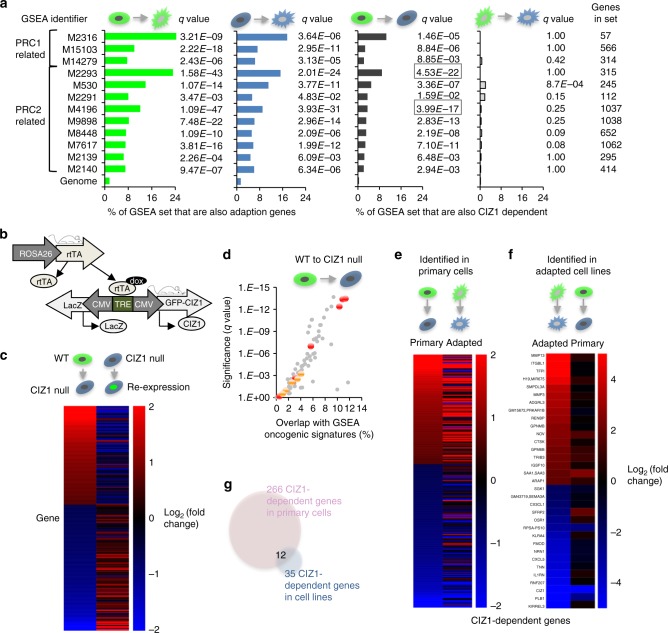
Relaxation of polycomb-regulated gene expression. **a** Relationship between affected transcription units (*q* < 0.05, cuffdiff) in Fig. 2 and PRC1 or PRC2-related curated gene sets (GSEA MSigDB^22^), with significance expressed as *q* value. Set identifiers and number of genes in sets are indicated. Overlap with genes affected by culture adaption of WT cells (green) and CIZ1-null cells (blue). Overlap with CIZ1-dependent genes in primary cells (dark grey) and culture-adapted cells (light grey). **b** Schematic of transgenes used to create doxycycline-inducible expression of full-length GFP-CIZ1 in CIZ1 null mice and derived cells^5^. **c** Heat map showing 266 transcription units (*q* < 0.05, cuffdiff) affected by the loss of CIZ1 in primary cells, organised by fold-change. Right column, mean fold change in the same transcription units in CIZ1 null cells (13.17 p3, 14.19p1), 24 h after induction of full-length CIZ1^5^ (Supplementary data 4, sheet 2). **d** Relationship between CIZ1-dependent transcription units in primary cells (*q* < 0.05, cuffdiff) and all 189 oncogenic signatures (GSEA MSigDB^22^), showing overlap (% of genes that are affected by the loss of CIZ1) against significance. Grey dots indicate oncogenic signatures not related to PRC 1 or 2. Red, sets that are up-regulated when a PRC subunit is down-regulated, or vice versa. Yellow, sets that are upregulated when a PRC subunit is upregulated or vice versa. **e** Heat map showing fold-change in 266 CIZ1-dependent transcription units (*q* < 0.05, cuffdiff) in primary cells (left, same as Fig. 1c), compared to fold-change in the same genes in culture-adapted derivatives, upon loss of CIZ1 (right). Supplementary data 4, sheet 3. **f** Heat map showing the 35 transcription units that are CIZ1-dependent in adapted cells (*q* < 0.05, cuffdiff) (left), and fold change in these transcription units in primary cells (right). Supplementary data 5. **g** Venn diagram showing overlap between transcription units that are differentially expressed upon loss of CIZ1 in primary and derived lines. Highlighted in Supplementary data 5. All *q* values (false detection rate corrected *P* values) for overlap with GSEA MSigDB were calculated using one-sided Fisher’s Exact tests with Benjamini–Hochberg false discovery rate correction. Heat maps are organised by fold-change from up (red) to down (blue)

As might be expected, transcriptome analysis revealed extensive differences between primary parent cell lines and their culture-adapted derivatives, with approximately a thousand genes significantly affected (1038 at *q* < 0.05 (cuffdiff), Fig. 2, Supplementary data 1). Similar results were achieved using primary and culture-adapted populations from CIZ1 null mice (904 at *q* < 0.05 (cuffdiff), Fig. 2, Supplementary data 1), approximately half of which (405) were the same genes as those affected in WT cells. For both sets, Gene Set Enrichment Analysis (GSEA)^22^ revealed significant overlap with genes regulated by PRC1 and PRC2 (Fig. 3a), as well as those responsive to Rb and TGFb among others (Supplementary data 2, tabs 4, 5, 6). PRC subunit expression was itself disrupted upon culture adaption, evident in both transcript level changes (Supplementary Fig. 2) and in splice variant diversity for some subunits (Supplementary Fig. 3, 4).

More informative is the effect of deletion of CIZ1 in primary cells. We showed previously that, despite enrichment at Xi, the loss of CIZ1 does not result in widespread reactivation of the inactive X-chromosome with only 28 X-linked genes significantly upregulated^5^. Here, we show that the majority of the 65,530 transcription units that align to the rest of mouse genome assembly GRCm38 are also unaffected (Supplementary data 3, Fig. 2). However, a sub-set of 266 transcription units (0.4%, *q* < 0.05 (cuffdiff)) consistently escape normal regulation in PEFs (Supplementary data 4, Fig. 2), with a similar proportion up- and down-regulated. Importantly, re-expression of full-length CIZ1 from an inducible integrated vector^5^ reverses this change back towards WT levels in 75% of affected genes within 24 h (Fig. 3b, c, Supplementary data 4) Thus, the expression of a distinct subset of genes is under the regulation of CIZ1 in primary fibroblasts, and these are distributed across all chromosomes, including the X. This is consistent with the interpretation that polycomb-mediated regulation is the primary pathway governing the expression of a subset of X-linked genes, and that for the majority inactivity is maintained by other levels of regulation.

GSEA with these CIZ1-dependent transcription units also revealed highly significant overlap with genes regulated by PRC1 and PRC2 (Fig. 3a, Supplementary data 2, tab 1), though unlike culture-adapted cells there was little change in PRC subunit transcript levels (Supplementary Fig. 2), and also no gross change in the overall immuno-staining pattern for SUZ12, RING1B or BMI1 in primary cells (Supplementary Fig. 2). Oncogenic Signature sets that are up-regulated upon the loss of PRC subunits, or down-regulated upon the gain of PRC subunits, show a strong correlation (Fig. 3d), while polycomb-linked Curated Gene sets also support a functional interaction, evidenced by highly significant relationships with those affected by manipulation of EZH2 (M4196^23^, *q* < 3.99E−17, where *q* is one-sided Fisher’s Exact test with Benjamini–Hochberg false discovery rate (FDR) correction,) or SUZ12 (M2293^24^, *q* < 4.53E−22) among others (Fig. 3a, Supplementary data 2 and Supplementary Tables 1 and 2).

Notably, a similar analysis of the effect of the loss of CIZ1 in culture-adapted populations returned only 35 CIZ1-dependent genes (*q* < 0.05, cuffdiff) and only 12 of these were among those identified in primary cells (Fig. 3e–g, Supplementary data 5). This is because many of those genes whose expression is normally dependent on CIZ1 are affected by culture adaptation of the WT state (39% *q* < 0.05, 56% *P* < 0.05). Again this argues that adaption to culture involves corruption of the process in which CIZ1 normally functions.

### Deletion of CIZ1 mimics the effect of culture adaptation

**Fig. 4:**
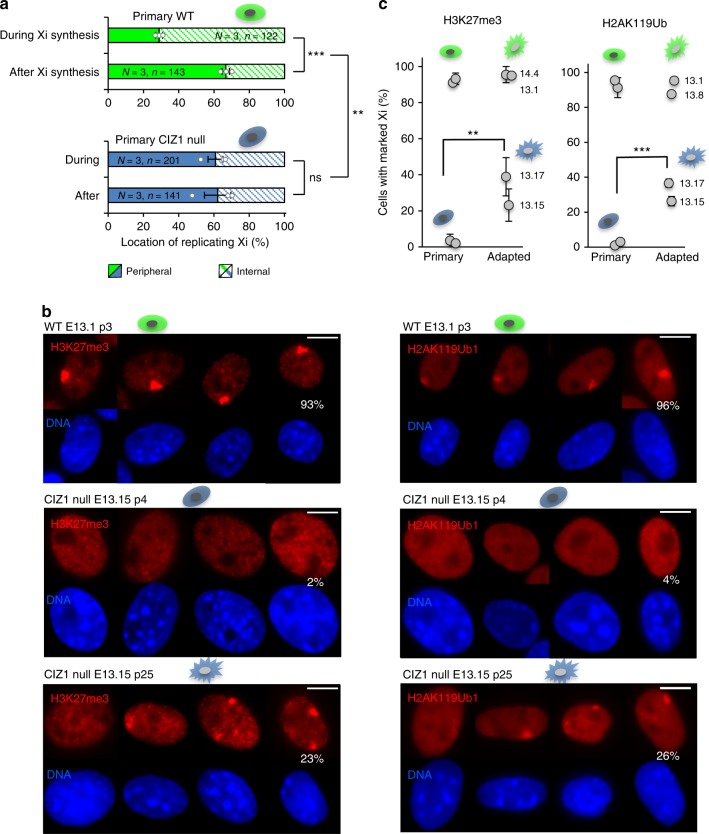
CIZ1 is required for PRC-mediated chromatin modification in primary cells. **a** Binary classification of Xi location (peripheral or internal) at the time of incorporation of EdU (pulse) or after (chase), analysed by *t*-test. Graph shows mean data ± SEM for three WT primary embryonic fibroblasts (green, 13.1, 13.8, 14.4 passage 2–3) and three CIZ1 null primary embryonic fibroblasts (blue, 13.15, 13.17, 14.2, passage 2–3). EdU-Xi in WT cells shifts preferred location during the chase period, but this remains unchanged in CIZ1 null cells, compared by ANOVA. *P ≤ 0.05, **P ≤ 0.01, ***P ≤ 0.001. *N* = independent cell lines, *n* = nuclei scored. Individual data points contributing to the mean are overlaid. **b** Representative immuno-stain of PRC1-mediated H2AK119Ub1 and PRC2-mediated H3K27me3 in primary WT cells (13.1, p3), absence of these marks in primary CIZ1 null cells (13.15, p4), and re-emergence in culture-adapted CIZ1 null MEFs (13.15, p25). Percentage of cells with marked Xis is shown. Scale bar is 10 microns. **c** Graphs show the proportion of primary and culture-adapted cells with H3K27me3-marked Xis (left) and H2AK119Ub-marked Xis (right), in CIZ1 null (blue) and WT (green) genotypes, ±SEM from triplicate analyses for each line. Significant changes upon culture adaptation are indicated, and compared by *t*-test

Because CIZ1 is highly enriched at Xi and undergoes specific interactions during Xi replication, we tested whether CIZ1 is required for Xi internalization in triplicate independent primary CIZ1 null populations. The data show that, unlike WT cells, Xi fails to change the preferred location within the test window, remaining primarily associated with the nuclear periphery in both the pulse and chase (Fig. 4a). It remains possible that a change in the *rate* of Xi movement, rather than complete loss of relocation, could account for the output in this assay. However, the data clearly show that CIZ1 plays a role in determining Xi location across the time window (and by implication the residency time at the nucleolus) thereby linking altered relocation with compromised polycomb function. Consistent with this, both PRC2-mediated H3K27me3^5^ and PRC1-mediated H2AK119ub1 are absent from Xi in primary CIZ1 null cells (Fig. 4b).

These data are consistent with the hypothesis that *accurate* receipt of polycomb-mediated chromatin marks is linked with the delivery of template into sub-nuclear compartments enriched in spatially restricted modifying enzymes^25^. In fact, EZH2, SUZ12 and Snf2h (part of the ACF1-ISWI chromatin remodelling complex involved in heterochromatin replication) have all been reported to be enriched at peri-nucleolar locations^25–27^. In our analysis, peri-nucleolar enrichment of EZH2 was also detected, but varied greatly with both cell type and the EZH2 antibody used (see later).

### Promiscuous polycomb activity in cell lines

Strikingly, enriched H3K27me3 and H2AK119ub1 re-emerges at Xi upon prolonged culture of CIZ1 null cells (Fig. 4b, c), sometimes describing a more dispersed Xi territory than in WT cells. This suggests that the deficit caused by the loss of CIZ1 is overridden during prolonged culture, and raises the possibility that similar compensation might also be taking place in WT cells (in which chromatin relocation is compromised for reasons unrelated to CIZ1). It could in fact underpin the relaxed control over polycomb target gene regulation illustrated in Fig. 3a, d, necessitating a direct comparison between the different cell states.

### CIZ1 deletion or culture adaptation drives a shift in EZH2

**Fig. 5:**
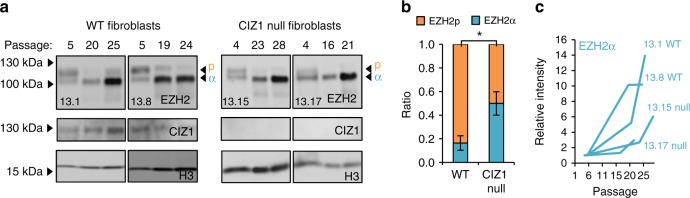
Loss of CIZ1 drives changes in EZH2 isoform. **a** Western blot showing PRC2 catalytic subunit EZH2 (antibody D2C9) in representative primary embryonic fibroblasts and derived culture-adapted populations, of WT and CIZ1 null genotypes, showing two prominent isoforms in early passage cells, designated p and α. Relative affinity for these isoforms varies between commercially available anti-EZH2 antibodies (Supplementary Fig. 3). **b** Quantification of isoforms p and α in early passage WT and CIZ1 null cells, showing mean ratio for three independent populations of each type (WT; 13.1, 13.8, 14.4, CIZ1 null; 13.15, 13.17, 14.2), ±SEM, significant changes are indicated (*t*-test). **c** Quantification of EZH2α in primary cells and derived populations for the cell lysates shown in **a**, after normalization to histone (H3) and calibration to the early passage population for each line. All four, irrespective of genotype, show increasing EZH2α with passage

Protein level analysis of the PRC2 catalytic subunit EZH2 resolved two main isoforms in primary cells, though relative levels vary between WT and CIZ1 null cells (Fig. 5a, b, Supplementary Fig. 3). The upper form (designated EZH2p for primary) that is dominant in WT cells gives way to a lower form as cells are adapted to growth in culture, and this is increasingly up-regulated with passage (Fig. 5a, c). EZH2 transcript assemblies also identified differences in prevalence (Supplementary data 6, Supplementary Fig. 3). The most abundant assembly in adapted cells corresponds to UniProt Q61188 (equivalent to human Q15910-1, designated EZH2α^28^), however this is vastly under-represented in primary cells and replaced by a form with additional 5ʹ sequence. No canonical translational start codon is evident in the additional sequence which is currently annotated as 5ʹ UTR. The relationship between EZH2p and the apparent 5ʹ UTR sequence that is prevalent in the same cells (Supplementary Fig. 3, Supplementary data 6) is not clear at this time. Electrophoretic mobility suggests that the lower protein isoform is equivalent to canonical EZH2α, so in primary fibroblasts EZH2 protein appears to include additional domains that are not part of the EZH2 that is expressed by cell lines. A similar analysis of transcript assemblies for the other subunits of both PRC1 and PRC2 identified additional changes linked with either deletion of CIZ1 or culture adaptation (Supplementary Fig. 4).

**Fig. 6:**
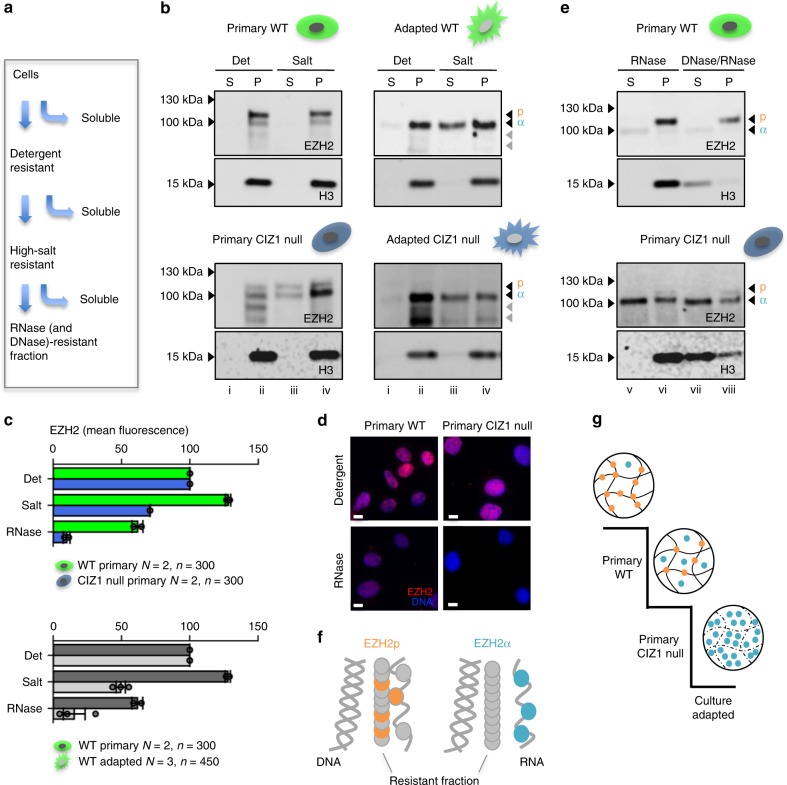
Loss of CIZ1 drives changes in EZH2 binding. **a** Schematic showing sequential extraction with detergent-containing buffer (0.1% Triton X100), high-salt buffer (500 mM NaCl), and nucleases (RNase and/or DNase1), detailed in Methods. **b** Representative western blots showing partitioning of EZH2p and EZH2α (antibody D2C9) into soluble (S) and pellet (P) fractions, after incubation with detergent (Det) or with detergent plus 500 mM NaCl (Salt). All fractions within an extraction series are cell-equivalents. Results are shown for primary WT (13.8 p3) and CIZ1 null cells (13.17 p5) and culture-adapted derived populations (p28–29) of each genotype. Fraction numbers i–iv relate to the full serial extraction protocol in Methods. **c** Quantification of EZH2 immuno-stain in two primary cell populations of WT (13.1, 13.8) and CIZ1 null (13.17, 14.2) genotypes after the indicated extraction steps. Histogram shows mean fluorescence intensity normalized to detergent-treated samples, ±SEM, where *n* > 50 for each genotype and condition, and *N* = 2. Below, the same analysis comparing two WT primary cells and three WT culture-adapted cells (13.1, 14.4, 3T3 cells). Individual data points contributing to the mean are overlaid. **d** Images show EZH2 (red, D2C9) after detergent-extraction and after RNase-extraction. DNA is blue, scale bar is 10 microns. **e** The salt-resistant pellet fraction (iv in **e**) from primary cells, was further partitioned by digestion to solubilize RNA, or both RNA and DNA as indicated, revealing complete nuclease-resistance of EZH2p in WT cells, despite efficient release of histone 3 (H3) into the soluble fraction. In contrast, in CIZ1 null cells (expressing EZH2α) more than half is sensitive to solubilisation with RNase. Fraction numbers v–viii relate to the full serial extraction protocol in Methods. **f** Illustration of the differing modes of attachment of EZH2α (released along with RNA), and EZH2p (immobilized by attachment to RNase and DNase-resistant assemblies). **g** Model illustrating two drivers of the shift away from immobilized EZH2p (primary WT cells) towards the expression of high levels of non-immobilized EZH2α (culture-adapted cells). CIZ1 null primary cells represent an intermediate state

The relative solubility of EZH2 in primary and culture-adapted cells point to function-related differences. Both EZH2p and EZH2α are fully resistant to salt extraction (500 mM) in primary cells, while half of the EZH2α in culture-adapted cells is solubilized under the same conditions (Fig. 6a, b), possibly reflecting elevated levels and saturation of binding sites. Similar results were obtained by quantitative immunofluorescence in independent WT primary cells and derived cell lines (Fig. 6c, lower graph). Thus EZH2 shifts isoform, is dramatically elevated and considerably more soluble in cell lines.

Focusing on the primary cell state, we applied further extraction steps (Fig. 6a, e) to reveal proteins that are solubilised along with fragmentation and elution of chromatin, those that are part of assemblies that are dependent on nuclear RNA, and those that resist all extraction. This highlights differences in EZH2 that are driven by the loss of CIZ1. Unlike previous analyses of EZH2 in embryonic stem cells^29^, the prevalent form in WT cells (EZH2p) does not depend on either RNA or DNA for retention in the nucleus. However, the prevalent form in CIZ1 null cells (EZH2α) is largely released by the removal of RNA (Fig. 6e, f). Consistent results were achieved by immuno-microscopy in two independent primary lines each for WT and CIZ1 null genotypes confirming distinct differences in extraction profile (Fig. 6c upper graph, d), and is in line with analyses of recombinant PRC2 which report promiscuous RNA-binding capability^30^. Thus, EZH2α lacks the ability to become anchored within the nucleus to non-chromatin, non-RNA protein assemblies (Fig. 6g).

**Fig. 7:**
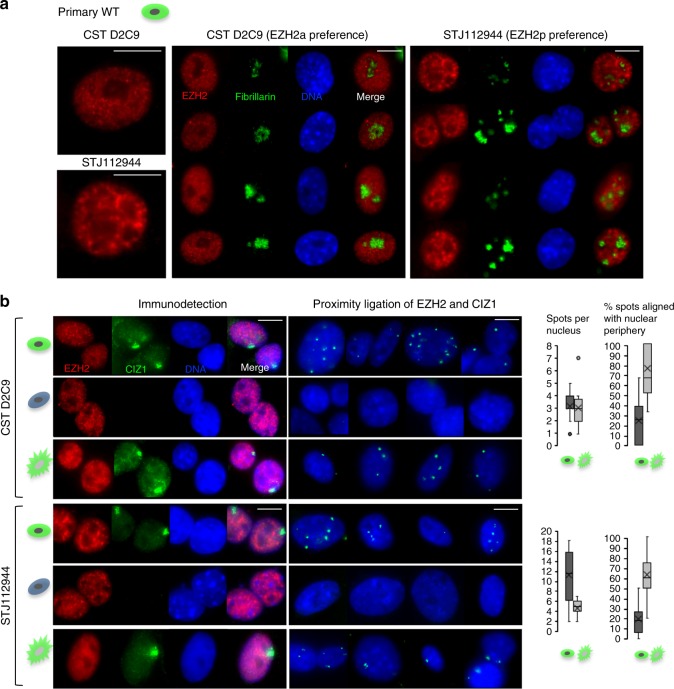
Differential isoform detection with EZH2 antibodies. **a** Immuno-detection of EZH2 with two different EZH2 antibodies (CSTD2C9 and STJ112944), and of the nucleolar protein fibrillarin in primary mouse embryonic fibroblasts (14.4 p3) showing distinct EZH2 staining patterns, and peri-nucleolar enrichment with STJ112944. Scale bar is 10 microns. **b** Immuno-detection (left) and proximity ligation (middle) of two different EZH2 antibodies (CSTD2C9 and STJ112944) with CIZ1 (hC221) in primary WT (14.4 p3), primary CIZ1 null (13.15 p2) and culture adapted WT (13.8 p23) embryonic fibroblasts. For EZH2 image intensity is differentially adjusted to reveal pattern rather than relative signal intensity. Scale bar is 10 microns. Right, quantification of proximity ligation assay showing the number of spots per nucleus (*n* = 20) and the percentage of spots aligned with the nuclear periphery for the primary and culture adapted WT cells, for both EZH2 antibodies. X represents the mean, the mid-line shows the median, the top and bottom line of the box the 3rd and 1st quartiles, and the whiskers the minimum and maximum values, excluding outliers (points, greater than 1.5 times the interquartile range)

Most of the analysis in this study was carried out using EZH2 antibody CSTD2C9 which recognises both EZH2p and EZH2α in western blots (Fig. 5a), and gives relatively evenly distributed nuclear foci by immunofluorescence in either primary or adapted cells before extraction (Fig. 6d, Supplementary Fig. 3). In contrast, EZH2 antibody STJ112944 has higher affinity for EZH2p than EZH2a in western blots (Supplementary Fig. 3) and reveals a distinctly different pattern in primary cells but not adapted cells (Supplementary Fig. 3). Comparison to the nucleolar marker fibrillarin identified this as peri-nucleolar enrichment (Fig. 7a), and is consistent with the idea that EZH2p represents a functionally distinct and spatially restricted form of EZH2 in primary cells.

Both EZH2 antibodies were also applied in proximity ligation studies with CIZ1, to reveal locations in the nucleus where the two proteins are in close proximity (Fig. 7b). We did not see a large signal comparable to the enrichment of CIZ1 at Xi with either antibody, in any cell type, or at any stage in the cell cycle, which would be consistent with a stochastic interaction between EZH2 and Xi chromatin. However in WT cells, but not CIZ1 null cells, both EZH2 antibodies return a signal at discrete locations in the nucleus, indicating proximity to within 40 nm of each other at those sites. In line with the western blot data in Fig. 5a, for EZH2p (antibody STJ112944), the number of colocalizing sites is reduced in adapted WT cells compared to primary WT cells. Moreover, consistent with the observation that CIZ1-Xi is compromised in its ability to internalize in adapted cells, fewer of these colocalizing sites are located internally in culture adapted cells compared to primary cells.

## Discussion

**Fig. 8:**
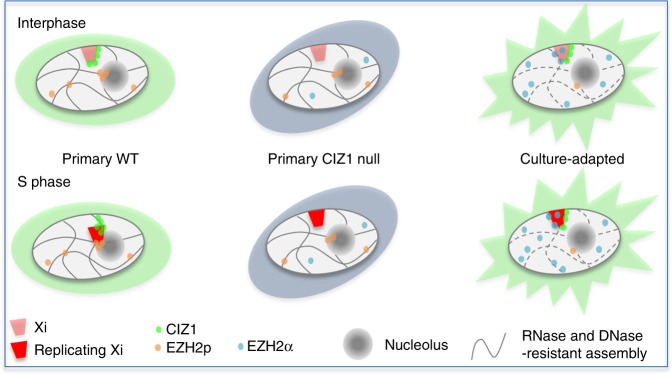
Summary model. Summary model depicting two alternative pathways by which chromatin and modifying enzymes could meet. Left, anchored and spatially restricted EZH2p (orange) in WT primary cells, and CIZ1-dependent relocation of Xi during replication of Xi. Centre, compromised relocation of Xi in CIZ1 null PEFs and loss of marks from Xi. Right, elevated wild EZH2α (blue) in culture-adapted cells is depicted diffusing to sites of action. We propose that this compensates for the loss of Xi relocation capability but could lower the stringency with which epigenetic state is maintained in cycling somatic cells

The data identify two drivers for the shift from EZH2p to EZH2α; the loss of CIZ1 in primary cells and culture-adaptation in both genotypes (Fig. 6g). Both drivers also result in the loss of observed S-phase location change and relaxed PRC-mediated control of gene expression (summary model, Fig. 8). We propose here that these parameters are linked, and suggest that soluble EZH2 that is released from spatial constraint may be a compensatory response to the loss of chromatin relocation capacity, enabling reinstatement of essential regulatory events, including the observed reinstatement of H3K27me3 at Xi in CIZ1 null cells. By analogy with our previous analysis of the solubility transition of cyclin E in human, mouse and Xenopus differentiation models^31^, we further suggest that unconstrained enzyme may represent reversion, during culture, to a less differentiated state.

Notably, in addition to a shift in solubility, culture-adapted cells also experience the elevation of EZH2 protein. Promiscuous activity and gain-of-function mutations in EZH2 are documented in a wide range of human cancers prompting interest in its potential as a therapeutic target^32^. The data presented here illustrate distinctions between EZH2 in normal cells in the body compared to EZH2 in transformed cells in culture, in terms of isoform, protein levels and solubility, which could inform design of selective interventions and should be considered in relation to the variable therapeutic response to inhibitors of EZH2^32^. Moreover, changes in polycomb function as a consequence of CIZ1 disruption, highlights a pathway by which the varied CIZ1-related diseases may arise. The switch in isoform expression, emergence of unconstrained wild enzyme and relaxed control over target selection that we show here, are consistent with the idea that compromised chromatin relocation may be a driver of epigenetic drift.

Moreover, the data strongly reinforce the message that culture-adapted fibroblast cell models do not fully represent the functional state of cells in the body, thereby restricting the application of somatic cell genetic modification strategies that include selection to derive clonal lines.

Long-range directional movement of chromatin has been studied in detail using tagged loci and live imaging approaches in CHO cells^33^ or transformed human cell lines^34^. The latter identified distinct nuclear substructures (nucleolus and nuclear periphery) that locally constrained the movement of a subset of loci but did not report evidence for transient relocation between those sites, while the former measured inducible unidirectional migration of an engineered locus from the periphery to the nuclear interior. This study, and others that focus on chromosome territory relocation during DNA repair^35^ or entry to quiescence^36^ in early passage human fibroblasts, have begun to implicate the chromatin-bound motor protein nuclear myosin 1 (NM1)^37^, though little mechanistic detail has yet been uncovered.

The data presented here implicate CIZ1 in a mechanism underpinning Xi relocation in S phase, however a number of observations argue that this function extends beyond Xi to other loci. These are the presence of focal-CIZ1 in both male and female nuclei^5^, similar expression profiles in both male and female differentiating ES cells^6^, and the genome-wide effect of deletion of CIZ1 on polycomb-regulated gene expression. It should be noted that the present analysis does not directly address whether polycomb-regulated genes elsewhere in the genome visit the nucleolus. However, the behaviour of Xi, which can be readily monitored via locally high concentrations of CIZ1, appears to be a valuable and tractable native context in which to unpick the requirements of long-range chromatin relocation events.

Crucially, capacity to relocate Xi was compromised in all of the cell lines we studied, precluding full mechanistic analysis of the role of CIZ1 in cell lines. However, because cell-cycle-dependent recruitment of Xi-CIZ1 to RNA-independent assemblies is retained in these cells, we can infer that any role that this interaction plays in Xi relocation is not sufficient on its own, and that the relocation process is likely compromised at another level. To understand the mechanism by which relocation is normally achieved we now need to (i) define the transient S-phase specific interaction between CIZ1 and the NM (chromatin and RNA-independent assemblies), and (ii) in cells that are competent to support an S phase shift in location of Xi, ask what role this plays in the maintenance of epigenetic landscape in daughter cells. An important caveat is that we cannot yet be certain that the impact on Xi location in CIZ1 null cells is direct or indirect, but the above approaches will help to establish the relationship.

A pivotal role for LNCRNAs in long-range movement of chromatin is suggested by disruption of *Xist*^25^ and *Firre*^38^ (which also results in the loss of H3K27me3 from Xi), and by studies unrelated to Xi, which implicate Kcnq1ot1 in perinucleolar targeting of an episomal vector during mid-S phase^39,40^. Again this was shown to contribute to silencing of linked genes by EZH2^41^. Thus, locus-specific relocation as a means of maintaining epigenetic state is an emerging function of LNCRNAs and is supported by independent studies. CIZ1 interacts with *Xist* via its repeat E and is dependent on repeat E for accumulation at Xi^5,6^, apparently through most of the cell cycle. The S-phase switch in CIZ1 interaction profile leads us to consider whether a primary function of CIZ1 may be to transiently link *Xist*-coated Xi chromatin to machinery that supports Xi transit in S phase, thereby coupling replication to relocation. Such an S-phase-specific function for CIZ1 is consistent with previous analyses which have identified direct and sequential interactions with cyclin E and cyclin A^42^, and regulatory phosphorylation sites which impact on CIZ1s function in DNA replication^43^, though at present are difficult to reconcile with data which suggest a link with pre-IC conversion^43^.

While the role of CIZ1 in determining Xi location in primary cells is beginning to emerge, the drivers that lead to the loss of S-phase location change in WT cells in long-term culture and the molecules affected, remain opaque. It could reflect changes in *Xist*^25^ or *Firre*^38^, or other chromatin-associated RNAs^44^, or a corrupted NM, or other factor such as NM1. However, all we can safely conclude at this stage is that corruption of the process in which CIZ1 normally functions is a recurrent feature of culture-adapted cells.

In summary, our data are consistent with the model that the selection of EZH2 target chromatin may be achieved by divergent pathways with differing fidelity. (i) A CIZ1-dependent, replication-linked pathway operating in normal WT somatic cells that supports timely and controlled meeting of spatially restricted enzyme and template via transient relocation of chromatin, and (ii) a CIZ1-independent pathway that is not dependent on chromatin relocation. Based on profiling the expression, location and binding characteristics of EZH2, we further hypothesize that release and up-regulation of EZH2 methyltransferase could compensate for the loss of relocation capability, enabling chromatin maintenance (at the expense of fidelity) in rapidly cycling cells. While the evidence for this last point is currently circumstantial, based on the data presented here, we are able to safely conclude that CIZ1 is part of a mechanism that contributes to accurate maintenance of epigenetic landscape in primary cells, and suggest that this pathway more closely reflects the status of normal differentiated cells in the body.

## Methods

### Cell isolation, culture and derivation of lines

CIZ1 null mice were generated from C57BL/6 ES clone IST13830B6 (TIGM) harbouring a neomycin resistance gene trap inserted downstream of exon 1. The absence of *Ciz1/*CIZ1 in homozygous progeny was confirmed by qPCR, immunofluorescence and immunoblot^5^. Mouse PEFs were derived from individual embryos at days 13–14 of gestation. All primary and culture adapted MEFs were cultured in DMEM GlutaMAX (Gibco) supplemented with 10% FBS (PAA) and 1% Pen/Strep/Glutamine (Gibco). During rapid proliferation phase, cells were grown to 90% confluence then passaged 1:3 every 2–3 days. Populations of cells cultured up to passage (p) 5 were considered early passage (specified in text), and designated here as primary cells. Experiments were not performed after passage 5, unless to generate culture adapted lines (Supplementary Table 3). To achieve this, beyond passage 5, cells were supplemented with fresh media every 3 days and passaged at 90% confluence (typically once per week as proliferation rate slowed) without dilution (passage ratio 1:1). By passage 10–12 individual colonies of intermediate state cells emerged, that were dissociated and replated as a whole (1:1) until rapidly proliferating culture adapted populations emerged by passage 20 and designated as culture adapted lines. These were routinely grown to 90% confluence then passaged as for primary cells. For inducible cells harbouring transactivator and responder transgenes, the addition of doxycycline to media (5–10 μg/ml) was used to induce GFP-CIZ1 within 6 h. Female 3T3 cell line D001^45^ (a kind gift from Stephen Downes) were grown in the same media.

### Ethics

All work with animal models is compliant with UK ethical regulations. Breeding and genetic modification of mice were carried out under UK Home Office license and with the approval of the Animal Welfare and Ethical Review Body at the University of Leeds. Analysis on cells and tissues derived from these mice was carried out with the approval of the Animal Welfare and Ethical Review Body at the University of York.

### Sub-nuclear fractionation

All buffers were made in RNase-free water. All reagents were from Sigma unless otherwise stated. All samples are named by the last treatment in the series that they received. Extraction for analysis by western blot^46^ is detailed below, and extraction for analysis by immunofluorescence microscopy^47^ was performed on cells grown on coverslips using the same buffers. Briefly, plates of adherent cells were washed twice in ice-cold PBS then twice in ice-cold cytoskeletal buffer (CSK: 10 mM PIPES/KOH pH 6.8, 100 mM NaCl, 300 mM Sucrose, 1 mM EGTA, 1 mM MgCl_2_, 1 mM DTT, 1 cOmplete™ Protease Inhibitor Cocktail per 50 ml) and drained at a shallow angle on ice for 2 min. Cells were scrape harvested and supplemented with 2 mM PMSF before addition of Triton-X-100 to 0.1% and, to samples not subsequently receiving RNase, vanadyl ribonucleoside complex (VRC) to 2.5 mM (NEB). Samples were mixed by pipetting, and after 1 min on ice, centrifuged at 1000 × *g*. The soluble fraction from this stage was designated detergent (det.) supernatant (S, sample i Fig. 6b). The pellet fraction was either denatured for analysis by SDS-PAGE (det. P, ii) or resuspended in 0.1% Triton-X-100 and 0.5 M NaCl in CSK and incubated on ice for 1 min before second centrifugation at 1000 × *g* (salt S, iii, and P, iv). Pellets to be further extracted were washed with digestion buffer (40 mM Tris/HCl, 10 mM NaCl, 6 mM MgCl_2_, 1 mM CaCl_2_, pH 7.9, supplemented 1/500 with RNaseOUT for RNase-free samples), before resuspension in fresh digestion buffer. Samples were then incubated at 30 °C for 1 h with gentle agitation in the presence of DNase1 (Roche 04716728001) at 0.3 U/μl, RNase (Roche 11119915001) 0.5 U/μl, or both DNaseI and RNase, or no enzyme (mock). Before final centrifugation, reactions were supplemented to 0.5 M NaCl for 5 min, then separated to yield pellets (P, vi and viii) and supernatants (S, v and vii). Pellets were resuspended in 1× denaturing buffer (2% SDS, 15% glycerol, 1.7% betamercaptoethanol, 75 mM Tris pH 6.8 with bromophenol blue), and supernatants supplemented with 4× denaturing buffer and heated to 95 °C with repeated vortexing to shear remaining nucleic acid. For extraction for analysis by immunofluorescence, coverslips were incubated with CSK supplemented with 0.1% Triton-X-100 and 2.5 mM VRC (CSK-D) for 1 min. Following CSK-D removal coverslips were either fixed with 4% paraformaldehyde (detergent sample) or incubated with CSK-D with 0.5 M NaCl (CSK-DS) for 1 min. For coverslips to be treated with RNase, VRC was left out from this step onwards. Coverslips were washed twice for 1 min with digestion buffer (supplemented with 2.5 mM VRC for non-RNase samples) before incubation for 1 h at 37 °C in digestion buffer with 2.5 mM VRC (High Salt sample), digestion buffer supplemented with 2.5 mM VRC and DNase1 at 0.45 U/μl (DNase sample) or digestion buffer supplemented with RNase at 0.75 U/μl (RNase sample). Coverslips were incubated for 1 min with CSK-DS (supplemented with 2.5 mM VRC for non-RNase samples) before fixation with 4% paraformaldehyde.

### Antibodies and detection protocols

For western blots, adherent cells were either extracted as above or, after washing in ice-cold PBS, scrape harvested into denaturing buffer with fresh 2 mM PMSF, to generate whole cell lysates. Samples were heated to 95 °C and separated through 4–15% gradient gels (Bio-Rad) then transferred onto nitrocellulose membranes using the iBlot system (Invitrogen). Blots were blocked in either 5% low-fat milk in PBS with 0.1% Tween-20, or 5% BSA in TBS with 0.05% Tween-20, depending on antibody, then incubated with primary antibodies overnight at 4 °C with gentle agitation. Blots were washed and probed with HRP-conjugated anti-species secondary antibodies for 1 h at room temperature (Jackson Immunochemicals 115-035-174 and 211-032-171), and imaged using EZ-ECL (Biological Industries) with a Syngene PXi chemiluminescence imaging system. Band intensities were quantified using GeneSys 4.03.05.0 software. Uncropped blots for the images used in main figures are shown in Supplementary Fig. 5. For immunofluorescence microscopy, cells grown on glass coverslips were washed with PBS before fixation in 4% paraformaldehyde, or where indicated bathed in CSK with 0.1% Triton-X-100 for 1 min prior to fixation. After fixation cells were rinsed twice with PBS then incubated for 15 min in BSA Antibody buffer (0.02% SDS, 0.1% TX100, 10 mg/ml nuclease-free BSA in PBS), followed by 2 h at 37 °C with primary antibody (in BSA antibody buffer). After three washes in the same buffer, anti-species secondary antibodies (Alexa Fluor 488 or 568) were applied for 1 h, followed by three further washes, and mounting in VectorShield with DAPI (Vector Labs). All antibodies including dilutions are listed in Supplementary Table 4.

### Proximity ligation assay

Primary WT, primary CIZ1 null and culture adapted WT murine embryonic fibroblast cells were grown on glass coverslips and washed with 0.1% Triton-X-100 containing PBS prior to fixation with 4% paraformaldehyde for 15 min. Coverslips were blocked and incubated with primary antibodies as usual before washing with Duolink^®^ In Situ Wash Buffer A and the proximity ligation assay detailed in Duolink^®^ PLA fluorescence protocol (Sigma), using species-specific secondary antibody probes and green detection reagents. In brief, following standard primary antibody incubation as detailed in antibodies and detection protocols, coverslips were washed twice for 5 min with Wash Buffer A and incubated with species-specific PLUS and MINUS PLA probes in the provided antibody diluent for 1 h at 37 °C. Coverslips were washed twice for 5 min with Wash Buffer A before incubation for 30 min at 37 °C with 1× Ligation Buffer supplemented with DNA Ligase. Coverslips were washed twice for 5 min with Wash Buffer A followed by incubation for 100 min at 37 °C in Amplification buffer supplemented with DNA polymerase. Coverslips were washed twice for 10 min with Wash Buffer B before a final 1 min wash in 0.01× Wash Buffer B and mounting in Vector Shield with DAPI (Vector Labs).

### Imaging

Fluorescence images were captured using a Zeiss Axiovert 200M fitted with a 63×/1.40 Plan-Apochromat objective and Zeiss filter sets 2, 10, 15 (G365 FT395 LP420, BP450-490 FT510 BP515-565, BP546/12 FT580 LP590), using Axiocam 506 mono and Axiovision image acquisition software (SE64 release 4.9.1). Where changes in fluorescence intensity are quantified across an extraction series, coverslips were imaged as a set with all images for each filter set captured with the same exposure time. Images were saved at 1499 by 1205 pixels in tagged image file format for downstream analysis. For EZH2 at least 50 individual nuclei from unmodified images were quantified for each cell population and each extraction condition, using Fiji^48^. Where indicated, EZH2 levels across an extraction series were normalized to the detergent-treated sample (given an arbitrary value of 100) to enable comparison between cell lines. For presentation, images were enhanced using Adobe Photoshop CS4 or Affinity Photo 1.5.2, maintaining identical manipulations across extraction series so that image intensities reflect actual relationships (unless specifically stated otherwise). All quantification of image intensity was carried out prior to manipulation. For the presentation of images illustrating positional, rather than intensity information, images were not necessarily modified identically.

### S phase labelling

5-Ethynyl-2ʹ-deoxyuridine (EdU, 10 μM) was added to adherent cells on coverslips at ~70% confluence for a 30 min pulse period under standard growth conditions. For pulse/chase experiments, coverslips were transferred to warm PBS then into fresh media (without EdU) for 30–60 min. To visualize newly synthesized DNA, coverslips were washed briefly in CSK with 0.1% Triton-X-100, or subjected to extraction up to the RNase treatment step before fixation with 4% paraformaldehyde for 15 min. Coverslips were then washed in PBS and EdU detected using the Click-iT® EdU Alexa Fluor® 594 kit (ThermoFisher), as recommended. Briefly, coverslips were blocked with 3% BSA before incubation in a light-proof humidified chamber with Click-iT® reaction cocktail for 60 min. For dual staining (e.g. CIZ1 or fibrillarin), coverslips were first incubated with primary antibody as described under antibodies and detection protocols before blocking with 3% BSA. Anti-species secondary antibody diluted in Click-iT® reaction buffer was included in the EdU detection step. Coverslips were then washed and mounted using VectorShield with DAPI (Vector Labs).

### Positional analysis of Xi

Typically, 30–50% of cells in a rapidly cycling population incorporate EdU during a 30 min pulse labelling period. Of these, 6–16% have one or two compact discrete patches of incorporated EdU against an early or mid S-phase replication pattern, verified as Xi by co-staining with CIZ1. The location of EdU-labelled Xi was scored in two ways. (i) By proximity to the nucleolar marker fibrillarin and the nuclear envelope, generating four categories illustrated in Supplementary Fig. 1. Xis were classified as peripheral if any part of its territory or individual foci that are part of a cluster were not resolvable from the nuclear perimeter, confirmed by comparison with DAPI. Xis were scored as nucleolar if any part of the territory or foci were unresolvable from fibrillarin stain. Xi which meets both of these criteria were classified as peripheral/nucleolar, or neither if both of the criteria were not met. (ii) A binary classification, based on the criteria above (Supplementary Fig. 1), was used to gather time-resolved data points. Xis were scored as internal if all of the territory, including all foci, was resolvable from the nuclear periphery, and peripheral if these criteria were not met. For nuclei with two EdU-labelled Xis both were classified individually. Nuclei with more than two EdU-labelled Xis were excluded from the analysis. At least 100 labelled nuclei were imaged per coverslip, with at least 3 replicate coverslips, and three biological replicate cell populations (from independent embryos) of each genotype. Results in Fig. 4a show compromised of Xi relocation in CIZ1 null cells, and results in Fig. 1f show a gradual diminishing of Xi relocation in WT adapted lines (evidenced by lack of change in the proportion of peripheral Xis between pulse and chase). However, in some adapted cells we were not able to score location because of the absence of a clearly defined CIZ1 mark and also apparent incoherent replication of Xi. In these cells, replicating Xi was no longer detectable as a patch of dense EdU against a mid-S phase nucleus, and neither was it identifiable against early or late replicating nuclei because CIZ1 was dispersed or absent. This most likely reflects instability of Xi in transforming cells, illustrated previously in the context of breast cancer^49^. These cell populations were typically late passage (p20 or more) and were excluded from the analysis.

### Statistical analysis

Experiments were designed to use the minimum number of animals while achieving statistically valid data and include two types of analysis. For multivariate data (lists of genes or proteins that are changed in one genotype compared to another) three biological replicates (independent PEF lines) enables calculation of average log 2 fold change and *T*-test *P*-values, as well as FDR-adjusted *P*-value for each data point (gene), and is the minimum required for differential expression analysis using Cuffdiff. Randomization is not appropriate, and PEF lines are matched as closely as possible. For single parameter endpoints such as analysis the requirements of Xi relocation, the variable is sampling time, and the output one of two positions, with typical effect size of 3 and analysis using two sample *t*-test in Excel. Unless indicated, data is represented as means with SEM. Comparison of the differing Xi relocation behaviour of WT and CIZ1 null cells was evaluated in R using a two-way ANOVA. *P < 0.05, **P < 0.01, ***P < 0.001. Avoidance of bias is achieved by independent, blinded analysis of archived images, for tests with optimised parameters. For example, if 1 mg/ml etoposide in the 30 min EdU chase period influences return of Xi to the nuclear periphery after replication, data (images) from replicate experiments will be archived, coded and blind scored by independent workers.

### Transcriptome analysis

Primary (before passage 5) and culture-adapted derivative cell lines of murine embryonic fibroblasts 13.1, 13.8 and 14.4 (female WT), and 13.15, 13.17, 14.2 and 14.19 (female CIZ1 null) were grown to 80% confluence. RNA was extracted with TRIzol (Ambion 15596-026) following manufacturer’s instructions. Briefly, adherent cells grown to 80% confluence were washed twice with PBS, drained on a shallow angle for 2 min and excess PBS removed. 1 ml of TRIzol was added per 28 cm^2^ and incubated for 3–5 min at room temperature with periodic agitation. Lysates were collected in clean Eppendorf tubes. Chloroform was added to a ratio of 1:5 and lysates were shaken vigorously for 15 s before incubation at room temperature for 3 min before centrifugation at 12,000 × *g* for 15 min. The aqueous phase was transferred into a clean Eppendorf and mixed with equal volume of isopropanol through gentle inversion and incubated at room temperature for 10 min, followed by a 10 min centrifugation at 12,000×*g*. Supernatant was removed, and the RNA pellet washed with an equal volume of 75% ethanol to the volume of TRIzol used to extract the RNA. Sample was centrifuged for 5 min at 12,000 × *g* and the supernatant removed. RNA pellet was resuspended in nuclease-free water. Isolated RNA was then treated with DNase (Roche 04716728001), before quality analysis by agarose gel, NanoDrop spectrophotometer and Agilent 2100 Bioanalyzer. Libraries were prepared with NEBNext® Ultra™ RNA Library Prep Kit for Illumina®, and enriched for mRNA using NEBNext Poly(A) mRNA Magnetic Isolation Module, which is optimized for the production of libraries with 250–400 bp inserts. Enriched mRNA was fragmented by heating to 95 °C for 12 min, cDNA synthesised from random primers, followed by end repair, dA-tailing, adaptor ligation and PCR enrichment. Libraries were sequenced at the Leeds Institute for Molecular Medicine (LIMM) using Illumina 3000 system, using paired-end sequencing to generate ~50 million reads per sample. Sequence reads were trimmed to remove any adapter sequences using Cutadapt version 1.8.3^50^ then aligned to version GRCm38 of the mouse genome using HISAT2^51^. Transcriptomes were assembled and gene expression quantified using the Tuxedo pipeline (version 2.2.1)^52^, from which predicted splice variant assemblies were extracted, including those that map to the EZH2 locus 6:47530040–47595351 (Supplementary Fig. 3, Supplementary data 6) and other PRC subunits (Supplementary Fig. 4). Average expression of each splice variant was calculated from the 3 independent lines for WT and CIZ1 null primary and culture adapted cells. Variants arising from sequencing of the opposite strand were removed. Total expression of the gene was calculated for each cell type along with the percentage of each splice variant. Splice variants contributing less than 5% of the total in all cell types were removed from the analysis to simplify visual representation. Cufflinks was used to assemble transcriptomes for each sample using the GTF annotation file for the GRCm38 mouse genome (*C57BL/6)*, followed by Cuffmerge to merge individual sample transcriptomes. Quantification, normalisation and differential expression were carried out using Cuffquant, Cuffnorm and Cuffdiff, respectively. GSEA^22,53^ was performed in Python 3.6 using one-sided Fisher’s Exact tests as part of the SciPy library (v.0.19.0). FDR was controlled using the Benjamini–Hochberg method in the StatsModels library (v.0.8.0), to generate *q* values. As this correction is based on *P*-values and different comparisons have different numbers of *P*-significant expression changes, the threshold at which *q*-significance is reached will vary between comparisons, as reflected in the volcano plots in Fig. 2. Volcano plots were generated in Excel. Heat maps were generated using Spyder (v.3.1.4), accessed via Anaconda Distribution (v.1.6.2), using the pandas, seaborn and matplotlib modules. Transcription units which did not have a numerical value for log_2_(fold change) due to mean expression of 0 in one condition were manually removed before generating the plots. Individual fragment counts per kilobase per million (FPKM) were extracted for replicate cell lines to calculate means and SEM.

### Reporting summary

Further information on experimental design is available in the Nature Research Reporting Summary linked to this article.

### Code availability

The custom code used to perform Gene Set Enrichment Analysis is available from authors upon request. All versions of software used are described in transcriptome analysis methods.

## Supplementary information

Supplementary Information

Description of Additional Supplementary Files

Supplementary Data 1

Supplementary Data 2

Supplementary Data 3

Supplementary Data 4

Supplementary Data 5

Supplementary Data 6

Reporting Summary

## Data Availability

Transcriptome data is available at GEO repository under accession code GSE122235. All other relevant data supporting the key findings of this study are available within the article and its Supplementary Information files or from the corresponding author upon reasonable request.
