# Supplementary material for: Maintenance of epigenetic landscape requires CIZ1 and is corrupted in differentiated fibroblasts in long-term culture
Source: Nat Commun. 2019 Jan 28;10:460. doi: 10.1038/s41467-018-08072-2 (PMC6484225; doi:10.1038/s41467-018-08072-2)
Supplement: Supplementary file 1 — Supplementary Information [file 41467_2018_8072_MOESM1_ESM.pdf]

## Supplementary information

# Maintenance of epigenetic landscape requires CIZ1 and is corrupted in differentiated fibroblasts in long-term culture

Stewart, Turner, Newling, Ridings-Figueroa, Scott, Ashton, Ainscough, Coverley

**Supplementary Figures and Legends**

**Supplementary Tables**

**References**

## Supplementary Figure 1

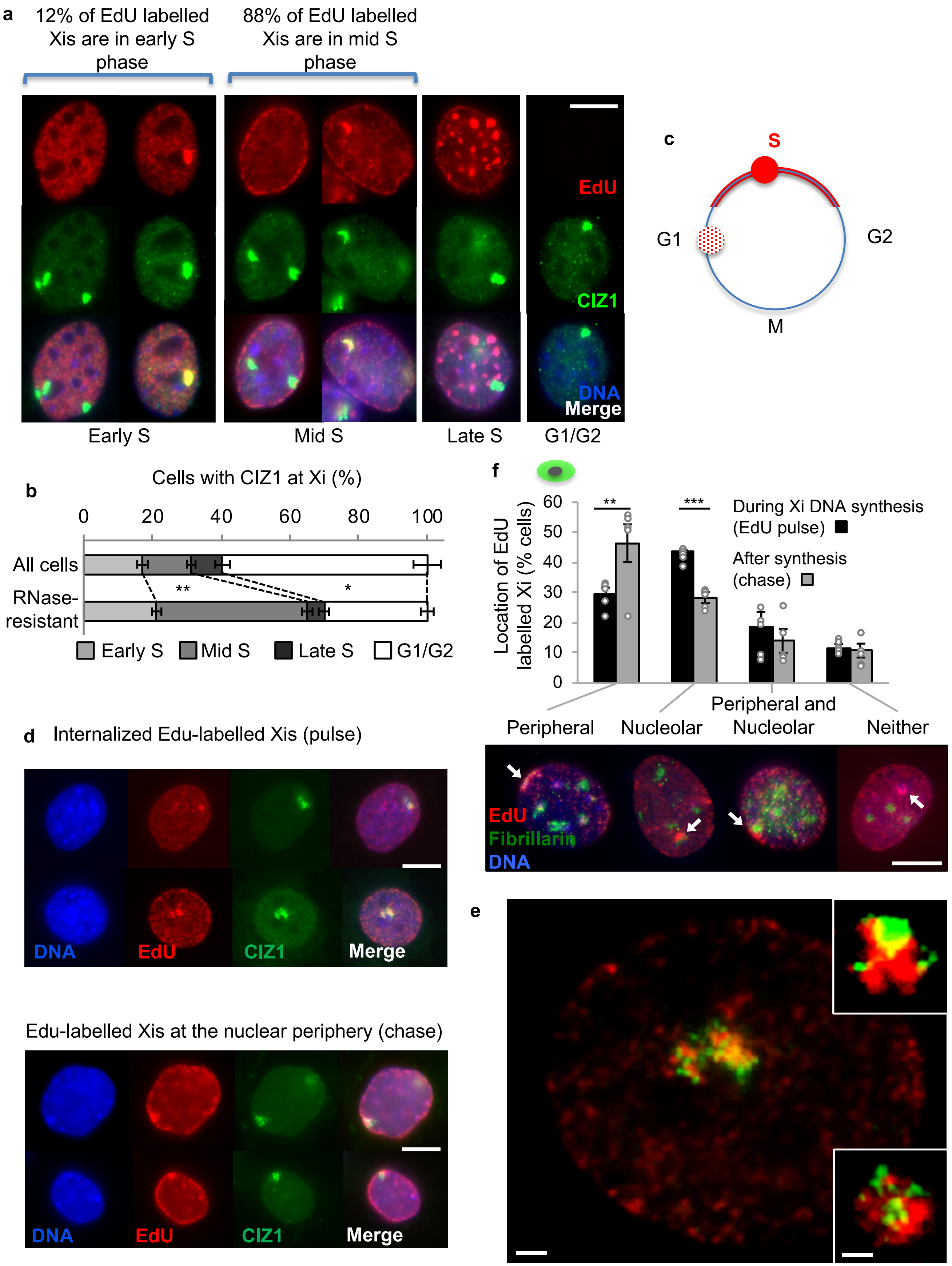

**Supplementary Fig.1 Location and timing of Xi replication**

a) Example images of 3T3 cell nuclei labelled with EdU (red) for 30 minutes immediately prior to isolation, classified as G1/G2 (no label), or early, mid or late S phase based on pattern of EdU incorporation. Scale bar is 10 microns. Immuno-staining for CIZ1 (green) reveals Xi, allowing estimation of the stage in S phase in which Xi incorporates EdU (given as %, n=100 replicating Xis).

b) Frequency of interphase (unlabelled) and S phase stages in nuclei with CIZ1 marked Xis, among a complete cycling population of 3T3 cells, compared to the frequency of each stage with RNase-resistant Xi-CIZ1. After RNase treatment preferential removal of Xi-CIZ1 occurs in G1/G2 phase, whereas preferential retention occurs in mid S phase. Data, ±SEM, is mean of 4 replicate analyses, n=1015, compared by t-test.

c) Schematic of mid-S phase window (red circle) in which CIZ1 at Xi is resistant to RNase. The data suggest there may be another window outside of S phase in which CIZ1 at Xi is resistant to RNase, possibly related to the early G1 phase window in which Xi has also been reported to transiently occupy an internalized location [1]. Notably, similar complicated positional changes, including an early G1 phase event followed by an S phase event, have also been reported for an engineered locus in CHO cells [2].

d) WT primary cells (13.1 and 13.8 p2-3) stained for EdU (red) and CIZ1 (green) showing example peripheral (chase) and internal (pulse) Xis. DNA is blue, bar is 10 microns.

(e) High-magnification views of three nuclei showing separation of the two signals within the Xi territory. Data relates to main Fig. 1. Scale bar is 1 micron.

f) Location of Xi at the time of its replication (EdU pulse, black) or after a 30min chase in EdU-free media (grey). Nuclear position, illustrated below, of EdU-Xi (red, arrowed) is classified in relation to the nuclear periphery and in relation to fibrillarin (green), to generate four categories. Histogram shows mean data (13.1 and 14.4) ±SEM, compared by t-test. Scatter plot of individual values contributing to the mean are overlaid. Bar is 10 microns.

**Supplementary Figure 2**

**a**

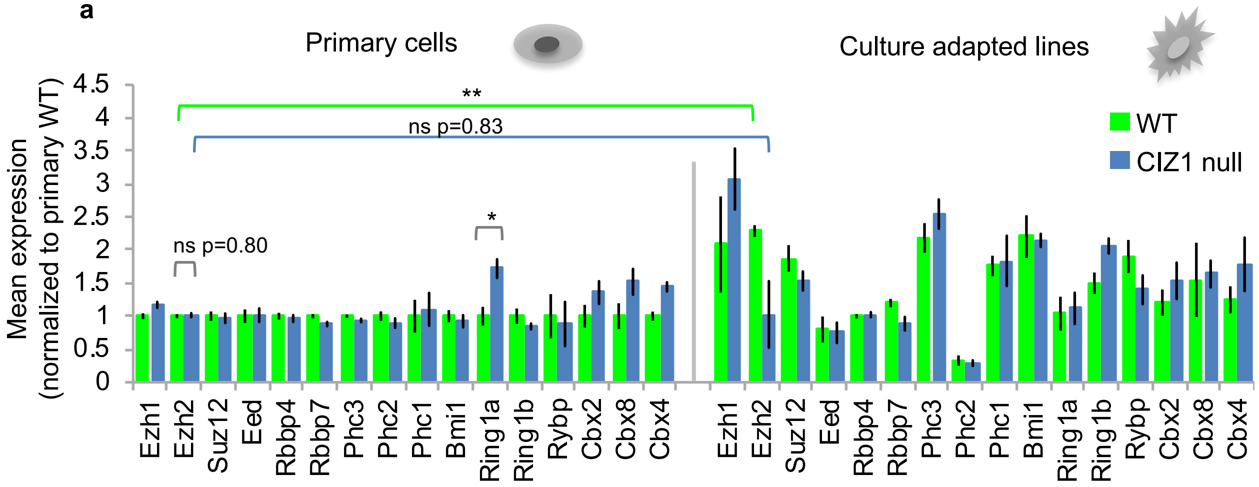


**b**

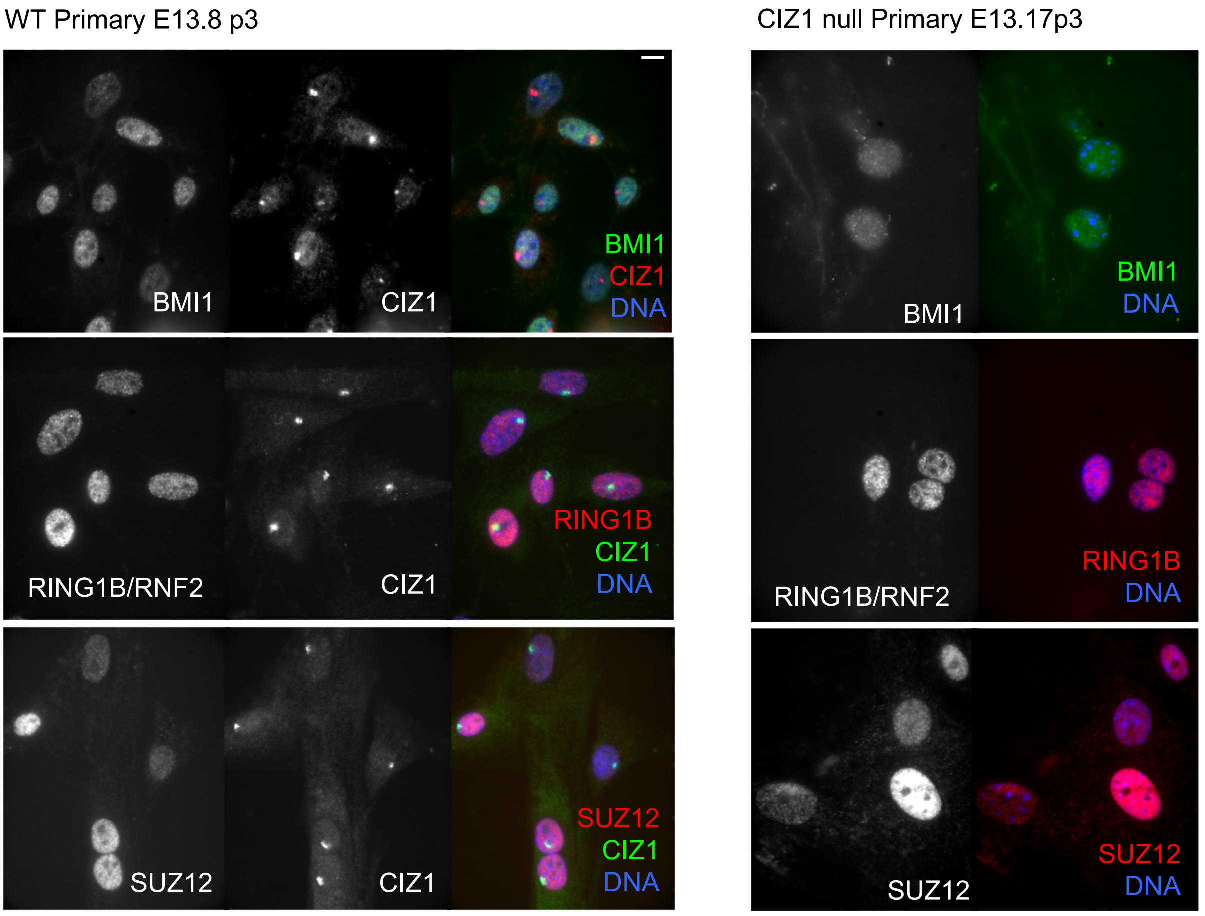


**Supplementary Fig. 2 Expression of polycomb subunits**

a) Mean transcript levels of PRC1 and PRC2 subunits, normalized to primary WT levels, ±SEM. Data is derived from FPKMs from three independent WT and CIZ1 null cell populations, showing little change in primary cells and inconsistent changes in culture-adapted derivatives, compared by t-test.

b) Immuno-detection of PRC1 subunits BMI1 (green) and RING1B (red), and PRC2 subunit SUZ12 (red) in primary WT and CIZ1 null cells. No gross differences in profile were observed, and no relationship with Xi-CIZ1. DNA is blue. Scale bar is 10 microns.

## Supplementary Figure 3

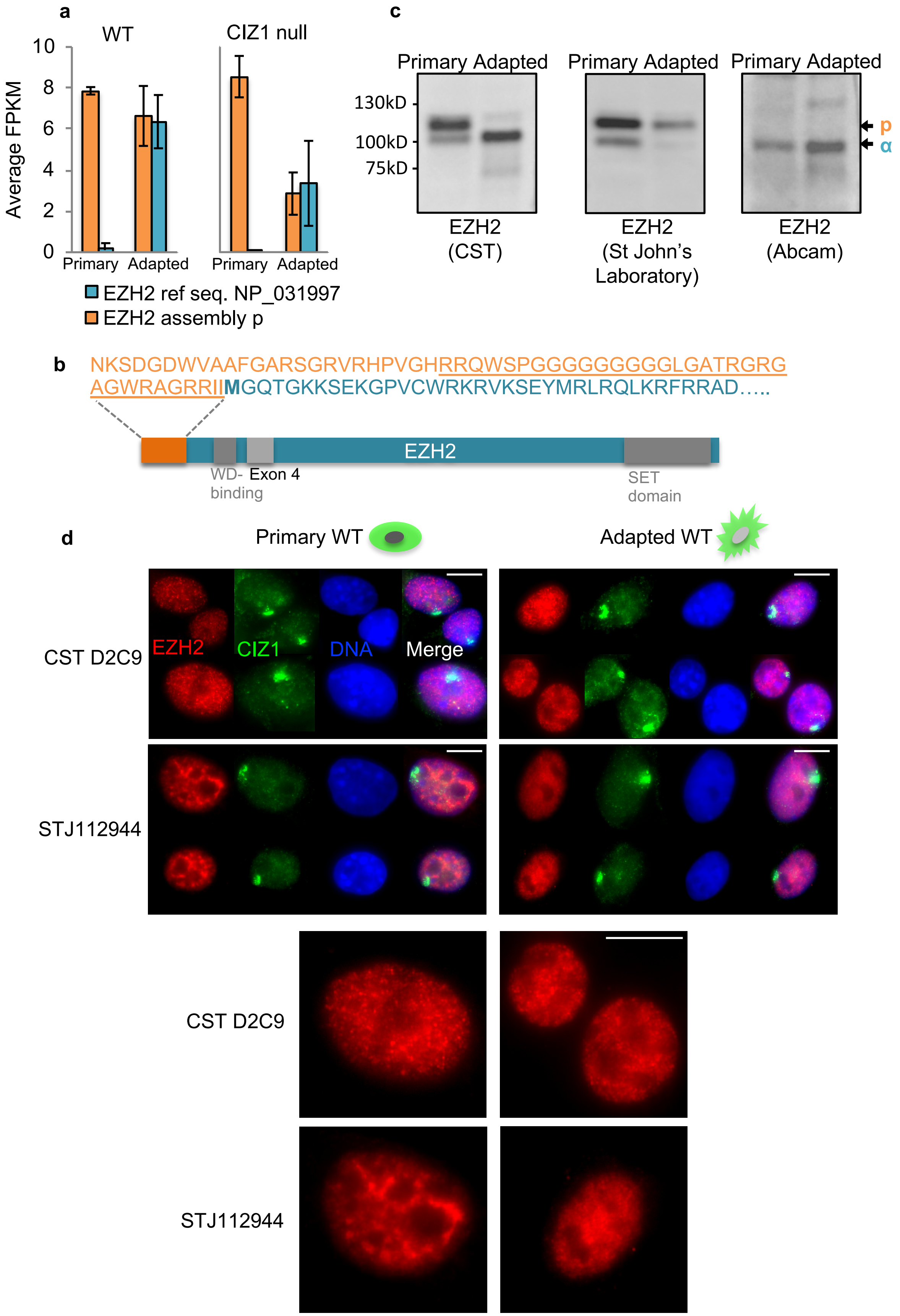

**Supplementary Fig. 3 EZH2 isoforms**

a) Splice variant assemblies derived from triplicate WT and CIZ1 null RNA-seq reads (primary cells and culture adapted derivatives) with mean frequency, ±SEM, for the two most abundant assemblies (Supplementary data set 6). These are canonical EZH2 (blue, which matches the reference sequence NP_031997), and a longer variant (orange, denoted p for primary) that includes additional 5′ sequence currently annotated as UTR but is otherwise identical (see Supplementary data set 6 sheets 2 and 3). Notably, canonical EZH2 is virtually undetectable in primary cells, while culture adapted cells express both variants at similar levels.

b) Translation of the upstream sequence in EZH2 assembly p gives rise to the putative sequence shown in orange. Canonical EZH2 begins with the M highlight in bold in blue. Approximately 50% of mammalian transcripts contain an upstream ORF (uORF), and many non-AUG triplets can act as alternative start codons for uORF translation [3].

c) Whole cell lysates from murine WT primary (p5) and culture-adapted cells (p21) showing dominant expression of EZH2p protein (upper form) in primary cells and EZH2$\alpha$ in later passage cells (lower form), detected with rabbit EZH2 antibody CST D2C9 (used in all main figures), which is centred on Arg354 of Q15910 (EZH2$\alpha$). EZH2p is also detected with rabbit EZH2 antibody STJ112944 (St John's Laboratory, unspecified epitope) but not rabbit EZH2 antibody ab191080 (Abcam), raised against a peptide epitope within amino-acids 1-100 of Q15910.

d) Immuno-detection of CIZ1 (hC221a) and EZH2 (CSTD2C9 and STJ112944) in primary (14.4, p3) and culture adapted (13.8, p23) WT murine embryonic fibroblasts. Below, high-magnification images. Scale bar is 10 microns.

## Supplementary Figure 4

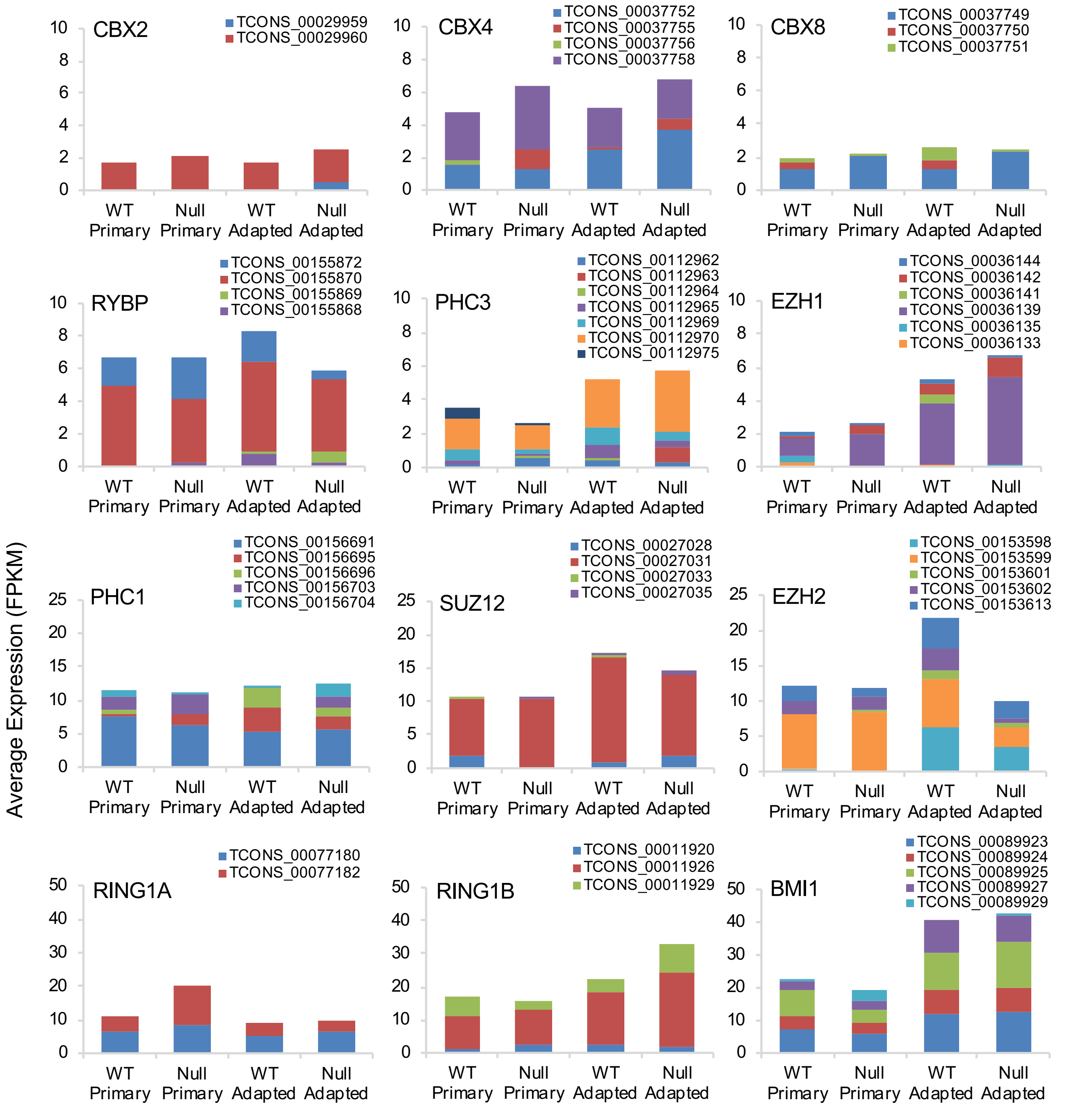


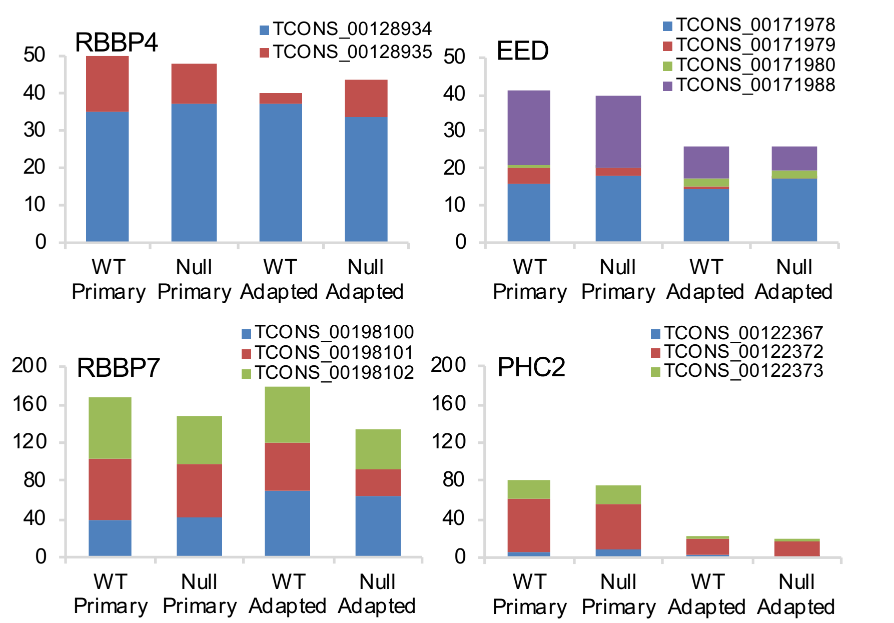


**Supplementary Fig. 4 Isoforms of Polycomb Repressive Complex Subunits**

Splice variant assemblies were derived from triplicate WT and CIZ1 null RNA-seq reads (primary cells and culture adapted derivatives). Histograms show the average total expression (FPMK) for each PRC subunit and the contribution of each splice variant (TCON).

## Supplementary Figure 5

Uncropped blots Figure 5a

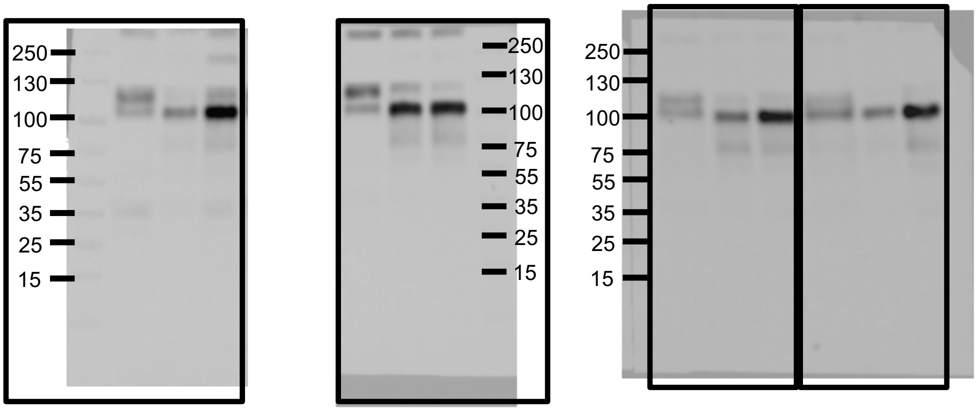


Uncropped blots Figure 6b

Uncropped blots Figure 6e

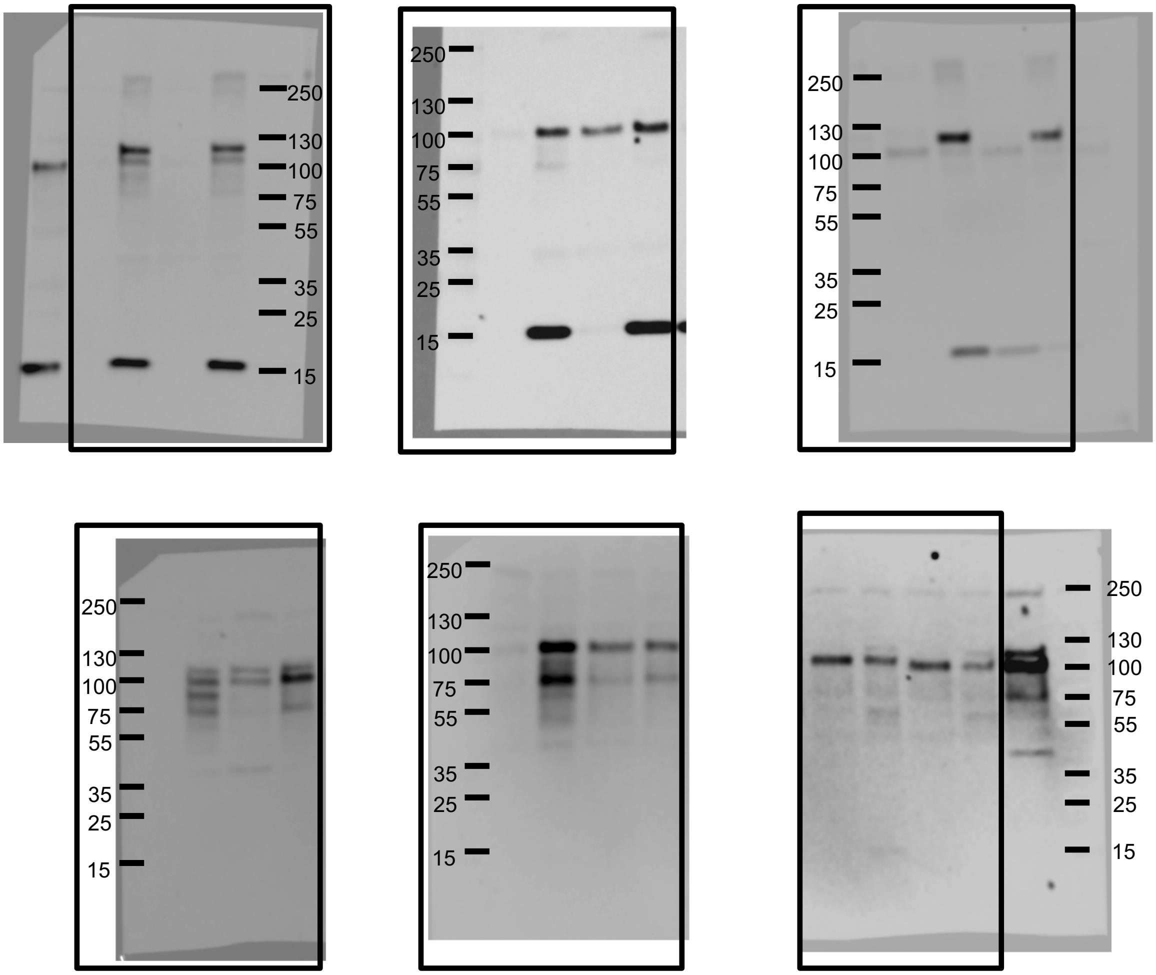


**Supplementary Fig. 5 Uncropped western blots** Images show whole western blots, with regions used in the main figures indicated by boxes. All are probed with EZH2 antibody CSTD2C9 generating bands between the 100-130 KDa markers, plus smaller forms in unextracted fractions. For blots on the middle row, histone H3 is also shown running slightly less far than the 15 kDa marker.

| Curated Gene Set | Percentage Overlap | p-value | q-value | Set Identifier used in Figure 3 |
|---|---|---|---|---|
| BENPORATH_EED_TARGETS | **2.730696798** | 1.40E-12 | **7.10E-11** | M7617 |
| BENPORATH_PRC2_TARGETS | **3.067484663** | 7.28E-10 | **2.19E-08** | M8448 |
| BENPORATH_SUZ12_TARGETS | **3.082851638** | 3.53E-15 | **2.83E-13** | M9898 |
| DOUGLAS_BMI1_TARGETS_DN | **2.229299363** | 0.001731182 | **0.00885055** | M14279 |
| DOUGLAS_BMI1_TARGETS_UP | **2.650176678** | 6.25E-07 | **8.84E-06** | M15103 |
| KONDO_EZH2_TARGETS | **4.897959184** | 1.61E-08 | **3.36E-07** | M5301 |
| LU_EZH2_TARGETS_DN | **2.173913043** | 0.000471418 | **0.00294496** | M2140 |
| LU_EZH2_TARGETS_UP | **2.372881356** | 0.00122352 | **0.00648217** | M2139 |
| NUYTTEN_EZH2_TARGETS_UP | **3.567984571** | 2.03E-19 | **3.99E-17** | M4196 |
| PASINI_SUZ12_TARGETS_DN | **8.888888889** | 9.59E-25 | **4.53E-22** | M2293 |
| PASINI_SUZ12_TARGETS_UP | **3.571428571** | 0.00347357 | **0.01585571** | M2291 |
| WIEDERSCHAIN_TARGETS_OF_BMI1_AND_PCGF2 | **10.52631579** | 1.10E-06 | **1.46E-05** | M2316 |

**Supplementary Table 1** PRC related gene sets from GSEA curated gene sets, represented in 266 transcription units significantly changed between WT and CIZ1 null primary cells

| **Oncogenic Signatures Gene Set** | **Percentage Overlap** | **p-value** | **q-value** |
|---|---|---|---|
| BMI1_DN_MEL18_DN.V1_DN | **4.081632653** | 0.000176213 | **0.00062838** |
| BMI1_DN_MEL18_DN.V1_UP | **10.34482759** | 1.08E-14 | **4.10E-13** |
| BMI1_DN.V1_DN | **3.472222222** | 0.001238695 | **0.00320703** |
| BMI1_DN.V1_UP | **10.88435374** | 6.63E-16 | **4.18E-14** |
| MEL18_DN.V1_DN | **4.054054054** | 0.000182553 | **0.00063894** |
| MEL18_DN.V1_UP | **11.34751773** | 3.63E-16 | **3.43E-14** |
| PRC1_BMI_UP.V1_DN | **2.604166667** | 0.00414869 | **0.00871225** |
| PRC1_BMI_UP.V1_UP | **1.052631579** | 0.270947659 | **0.32005692** |
| PRC2_EED_UP.V1_DN | **0.518134715** | 0.644300614 | **0.68029506** |
| PRC2_EED_UP.V1_UP | **1.546391753** | 0.087816979 | **0.11446489** |
| PRC2_EZH2_UP.V1_DN | **5.670103093** | 1.55E-08 | **1.04E-07** |
| PRC2_EZH2_UP.V1_UP | **2.564102564** | 0.004421212 | **0.00898212** |
| PRC2_SUZ12_UP.V1_DN | **3.141361257** | 0.000677083 | **0.00206401** |
| PRC2_SUZ12_UP.V1_UP | **1.030927835** | 0.278785281 | **0.32128304** |

**Supplementary Table 2** PRC related gene sets from GSEA oncogenic signatures, represented in the 266 transcription units significantly changed between WT and CIZ1 null primary cells

| Name | Sex | Type | CIZ1 genotype | Reference |
|---|---|---|---|---|
| E13.1 | Female | Primary embryonic fibroblast | +/+ | [4] |
| E13.8 | Female | Primary embryonic fibroblast | +/+ | [4] |
| E14.4 | Female | Primary embryonic fibroblast | +/+ | [4] |
| E13.15 | Female | Primary embryonic fibroblast | -/- | [4] |
| E13.17 | Female | Primary embryonic fibroblast | -/- with inducible CIZ1 transgene | [4] |
| E14.2 | Female | Primary embryonic fibroblast | -/- | [4] |
| E14.19 | Female | Primary embryonic fibroblast | -/- with inducible CIZ1 transgene | This paper |
| E13.1 | Female | Adapted fibroblast cell line | +/+ | This paper |
| E13.8 | Female | Adapted fibroblast cell line | +/+ | This paper |
| E14.4 | Female | Adapted fibroblast cell line | +/+ | This paper |
| E13.15 | Female | Adapted fibroblast cell line | -/- | This paper |
| E13.17 | Female | Adapted fibroblast cell line | -/- with inducible CIZ1 transgene | This paper |
| E14.2 | Female | Adapted fibroblast cell line | -/- | This paper |
| D001 3T3 | Female | Adapted murine fibroblast | +/+ | [5] |

**Supplementary Table 3** Cell Lines

| Antibody | Concentration | | Western Blocking Buffer | Company/Reference |
|---|---|---|---|---|
| | Western | IF | | |
| CIZ1 | 1:1000 | 1:1000 | PBS | 1794[6] |
| CIZ1 | | 1:20 | | hC221a |
| Fibrillarin | | 1:100 | | ab4566, Abcam |
| H2AK119Ub1 | | 1:1500 | | D27C4, CST |
| H3K27me3 | | 1:2000 | | ab6002, Abcam |
| EZH2 | 1:1000 | 1:250 | TBS | D2C9, CST |
| EZH2 | 1:1000 | | TBS | ab191080, Abcam |
| EZH2 | 1:1000 | | TBS | STJ112944, St John's Laboratory |
| H3 | 1:10000 | | PBS | ab1791, Abcam |
| BMI1 | | 1:500 | | ab14389, Abcam |
| RING1B | | 1:500 | | D22F2, CST |
| SUZ12 | | 1:500 | | D39F6, CST |

**Supplementary Table 4** Primary antibodies used for western blot and immunofluorescence.

**References to supplementary information**


1 Lyu, G. *et al.* Changes in the position and volume of inactive X chromosomes during the G0/G1 transition. *Chromosome Res* **26**, 179-189, 2018.

2 Tumbar, T. & Belmont, A. S. Interphase movements of a DNA chromosome region modulated by VP16 transcriptional activator. *Nat Cell Biol* **3**, 134-139, 2001.

3 Lee, S. *et al.* Global mapping of translation initiation sites in mammalian cells at single-nucleotide resolution. *Proc Natl Acad Sci U S A* **109**, E2424-2432, 2012.

4 Ridings-Figueroa, R. *et al.* The nuclear matrix protein CIZ1 facilitates localization of Xist RNA to the inactive X-chromosome territory. *Genes Dev* **31**, 876-888, 2017.

5 Coverley, D., Laman, H. & Laskey, R. A. Distinct roles for cyclins E and A during DNA replication complex assembly and activation. *Nature Cell Biology* **4**, 523-528, 2002.

6 Coverley, D., Marr, J. & Ainscough, J. Ciz1 promotes mammalian DNA replication. *Journal of Cell Science* **118**, 101-112, 2005.
