# Supplementary material for: Maintenance of epigenetic landscape requires CIZ1 and is corrupted in differentiated fibroblasts in long-term culture
Source: Nat Commun. 2019 Jan 28;10:460. doi: 10.1038/s41467-018-08072-2 (PMC6484225; doi:10.1038/s41467-018-08072-2)
Supplement: Supplementary file 2 — Description of Additional Supplementary Files [file 41467_2018_8072_MOESM2_ESM.pdf]

Legends for Supplementary Data files

Supplementary data set 1
Excel file showing mean data with significance indicators for all transcription units affected by culture adaptation ($q<0.05$) in WT cells. On sheet 2, similar analysis for CIZ1 null cells. Relates to Fig.2.

Supplementary data set 2
Excel file showing top 50 curated gene sets and top 50 oncogenic signature sets returned by GSEA with the indicated gene lists and sub-lists (11 tabs). Enrichments that meet the significance threshold $q<0.0005$ are highlighted in green, and PRC1/2 related gene sets in yellow.

Supplementary data set 3
Excel file showing whole transcriptome data for 3 WT and 3 CIZ1 null primary cell lines, plus mean data with significance indicators. On sheet 2, individual and mean data for derived culture adapted cell lines. Relates to Fig.2.

Supplementary data set 4
Excel file showing mean data with significance indicators for all transcription units affected by loss of CIZ1 ($q<0.05$), used in GSEA. On sheet 2, induction of CIZ1 in two CIZ1 null primary cell lines for genes affected by loss of CIZ1 in primary cells. On sheet 3, expression of CIZ1-dependent genes identified in primary cell lines, in adapted derivatives. Relates to Fig.3.

Supplementary data set 5
Excel file showing mean data with significance indicators for all transcription units affected by loss of CIZ1 ($q<0.05$), in culture-adapted cell lines. On sheet 2, expression of the same set of CIZ1-dependent genes in primary cells. CIZ1-dependent genes common to primary and adapted cells are highlighted. Relates to Fig.2.

Supplementary data set 6
EZH2 transcript assemblies with frequencies, derived from pooled reads mapping to locus 6:47530040-47595351, from triplicate WT and CIZ1 null cell lines of primary and adapted status, including graphical representation of data. On sheets 2 and 3, sequence of TCONs 00153599 and 00153598. Relates to Supplementary Fig.3.
